# Make it flow from solid to liquid: Redox-active electrofluids for intrinsically stretchable batteries

**DOI:** 10.1126/sciadv.adr9010

**Published:** 2025-04-11

**Authors:** Mohsen Mohammadi, Saeed Mardi, Jaywant Phopase, Filippa Wentz, Jibin J. Samuel, Ujwala Ail, Magnus Berggren, Reverant Crispin, Klas Tybrandt, Aiman Rahmanudin

**Affiliations:** ^1^ Laboratory of Organic Electronics, Department of Science and Technology, Linköping University, 601 74, Norrköping, Sweden.; ^2^ Wallenberg Wood Science Center, Department of Science and Technology, Linköping University, 601 74 Norrköping, Sweden.; ^3^ Ångström Laboratory, Department of Chemistry, Uppsala University, 751 21 Uppsala, Sweden.; ^4^ Wallenberg Initiative Materials Science for Sustainability, Department of Science and Technology, Linköping University, 601 74 Norrköping, Sweden.

## Abstract

High-capacity stretchable batteries are crucial for next-generation wearables to enable long-term operation and mechanical conformability with the human user. In existing stretchable battery designs, increasing the active material to yield higher capacity often leads to thicker and stiffer solid electrodes with poor mechanical properties. Here, we present a concept that transfers the physical property of a battery electrode from a conventional solid into a fluid state. The mechanical and electrochemical properties of the electrode rely on the viscosity of fluids rather than Young’s modulus of solids. Fluids conform easily into any shape with minimal force, making them intrinsically deformable. This decouples the electrochemical and mechanical property of the redox-active electrofluid, leading to higher capacities with more active material loading without stiffening the cell. The cell showed excellent capacity retention over 500 charge-discharge cycles and mechanical robustness up to 100% strain. Our work provides a technological solution for stretchable batteries that balances capacity and mechanical performance.

## Teaser

A stretchable battery electrode design uses fluids instead of solids to tune its electrochemical and mechanical properties.

## INTRODUCTION

Next-generation wearables are distinct from their commercial counterparts (e.g., earphones, hearing aids, smart watches, or fitness trackers) and require mechanical compliance with the human body that extends beyond flexibility to softness and stretchability (*1*). They have intricate form factors such as electronic (e)–skin patches, internal e-implants on the organs or nerves, e-textiles, and soft robots. Advancements have been made to enable suitable electromechanical properties of various device components (sensors, transistors, antennas, conductive tracks, etc.) in next-generation wearables (*2*, *3*). However, to power them, rigid and bulky batteries are used, which limits their form factor and mechanical compliance (*4*). For future wearables to have autonomous operation and energy demanding functions such as wireless data transmission, long-term sensor monitoring, and data logging, an integrated stretchable battery is required (*5*). With a forecasted trillion internet-of-things devices by 2035 (*6*), of which many will be wearables, the development of stretchable batteries is crucial in revolutionizing technologies for health care, environmental and food monitoring, and communication and entertainment.

**Fig. 1.**
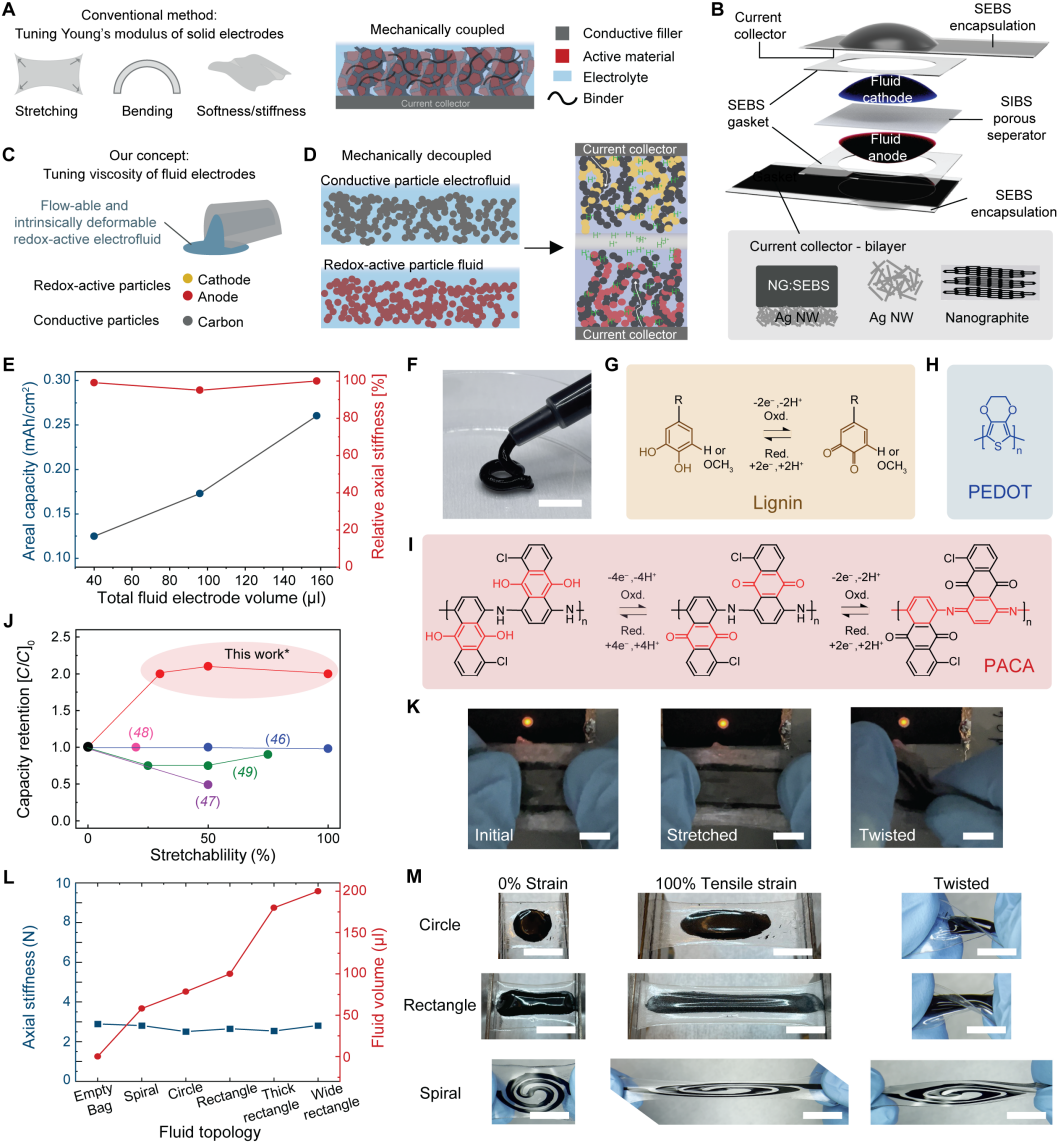
Redox-active electrofluid stretchable battery concept. (**A**) Conventional active electrode design paradigm. (**B**) Schematic of the battery components and the materials used. (**C**) Fluid electrode design concept. (**D**) Mechanically decoupled suspension fluids. H^+^ (Green) indicates protons in the electrolyte. The white arrows show the electronic conduction path along the carbon particles. (**E**) Relationship between total redox-active fluid loading of the cathode and anode (bottom), and cell capacity (blue, left) and axial stiffness (red, right). (**F**) Photograph of a representative electrode fluid extruding from a syringe. Scale bar, 8 mm. Molecular structure and redox processes of (**G**) modified lignin (L). R represents the heterogeneous structure of lignin, (**H**) conducting polymer, poly(3,4-ethylenedioxythiophene) (PEDOT) (P) and (**I**) poly(1-amino-5-chloroanthraquinone) (PACA). (**J**) Comparison of the capacity retention and stretchability of state-of-the-art fluid-based stretchable batteries. (**K**) Photograph of an assembled full cell in its initial, stretched and twisted state powering an LED. Scale bars, 10 mm. (**L**) Relationship between fluid topology (bottom) and relative axial stiffness measured at 20% strain (blue, left) and fluid volume (red, right). Empty bags refer to the encapsulation layer without the fluid. (**M**) Representative images of the different fluid topologies under different mechanical deformation. From left to right: 0% strain, 100% tensile strain, twisted. Scale bars, 10 mm.

Existing stretchable battery designs face a critical limitation in increasing capacity because adding more active material will lead to stiffer and thicker electrodes with poor mechanical compliance and stretchability (*7*, *8*). Fundamentally, they have adopted electrode designs from conventional rigid batteries that rely on the mechanical coupling (solid-to-solid contact) of the redox-active species and the conductive filler (Fig. 1A). A coupled electrode requires a binder to provide mechanical integrity in the solid matrix by holding the respective components together. This ensures good electronic contact between the components that facilitates access to the active material for efficient charging and discharging (*9*). However, any loss of contact between the components due to mechanical stress leads to a loss in battery operation. While the binder can be replaced with an elastomer to embed stretchability in the electrode (*10*), higher active material loading requires more conductive fillers and insulating binders. The thicker layers will experience higher strain during bending and larger forces to deform, which negatively affects mechanical compliance (*7*, *8*). Furthermore, thicker electrodes tend to have higher electrical resistance and tortuosity that hinder electrical and ion transport, limiting access to the active species in the solid electrode, resulting in a lower effective volumetric capacity (*11*, *12*).

Designing a stretchable battery requires a holistic approach that considers all components (active electrodes, separator, current collector, and encapsulation, Fig. 1B) (*7*, *13*) There are two general approaches to enable stretchability, structural engineering and material design. Structural engineering methods isolate rigid components from major strain within the system through various approaches, including the formation of layered twisted fibers of each battery component (*14*), connecting rigid islands of stiff battery cells onto deformable substrates with stretchable interconnects (*15*, *16*), sliding rigid electrodes (*17*), spiral (*18*) and wavy/buckled structures (*19*), kirigami (*20*, *21*), and ultrathin structures (*22*). Stretchable materials can be designed by mixing rigid active fillers with an elastomer to form composites (*10*, *23*–*27*), porous structures (*28*, *29*), and gels (*30*). Despite recent advancements, these methods are based on optimizing the conventional coupled electrode design that does not address the fundamental issue of the trade-off between battery capacity and mechanical properties (compliancy and stretchability).

Fluids have no fixed shape and yield easily to external pressure, making them intrinsically “stretchable” or highly deformable (*31*). Inspired by this fundamental behavior, we demonstrate that by transferring the physical property of the battery electrode from a conventional solid to a fluid state, it provides us with an electrode design concept that relies on viscosity of a fluid rather than the Young’s modulus of a solid (Fig. 1C). This decouples the electrochemical and electrical function from the mechanical property of the electrode, leading to a redox-active electrofluid that can conduct ions and electrons to store energy while maximizing deformability and capacity. Electrofluids consisting of conductive particles suspended in a non-conductive liquid or viscoelastic liquid-like matrix that forms dynamic electronic percolation networks have been reported (*32*, *33*). Here, we introduce redox-active electrofluid electrodes that are suspensions of redox-active and conductive particles in an electrolyte system (Fig. 1D).

At rest (static), the particles self-agglomerate into transient electrical and mechanical percolating networks, i.e., contact points. Under applied mechanical strain, they reversibly break and reform and flow dynamically as a fluid, alleviating mechanical stress while maintaining percolation. Because the particles are suspended in the electrolyte, viscosity is used to control the fluid’s mechanical properties, while energy storage is facilitated by electronic and ionic transport. Combining such an approach with other key components of a stretchable battery cell has several advantages, especially when interfacing with the body of mammals, which is based on the combination of solid and fluid states (*34*). Moreover, the fluid nature of the electrodes could enable printed batteries with form factor–free configurations that are well suited for next-generation wearables (*35*).

While fluids are widely used in electrochemical energy storage systems, they are designed for large-scale stationary batteries that require high volume storage tanks and pumps to flow the cathodic and anodic fluids reversibly through a current collector. This includes redox-flow batteries that involve an aqueous solution containing dissolved redox-active ions (*36*) and semi-solid flowable carbonaceous slurry electrodes with dispersed solid redox-active particles (*37*). Interesting methods using fluidic electrolytes containing dissolved molecular redox-species driven by capillary forces (*38*, *39*) or via a “plant-like” evaporation-induced diffusion have also been demonstrated (*40*). In the context of portable and wireless wearable devices, the physical (size and weight) and mechanical (stretchability and compliancy) properties are key design factors for stretchable batteries.

So far, only a handful of reports have exploited fluids such as liquid metals, either gallium or EGaIn, a eutectic alloy of gallium and indium. In these cases, the redox reaction is the reversible stripping/deposition of the gallium anode (see table S1 for a summary of prior work). Gallium, however, comes with several issues. It can only be used as an anode material, its hydrophobic surface impedes reaction activity with the hydrogel electrolyte, and it has the risk of converting into a solid (oxide) phase during the discharge, which hinders the redox reaction and decreases its mechanical compliancy, i.e., losing its fluid nature (*41*–*44*). Gallium-based liquid metals have shown promise as soft hermetic layers in the encapsulation to protect the battery (*45*). MnO_2_-carbon slurry electrodes have been reported either paired with a liquid metal anode (*46*) or with a Zn-carbon slurry (*47*–*49*), but their charge-discharge cyclic stability was poor, possibly due to the choice of battery electrolyte, making them prone to parasitic side reactions, and their mechanical robustness under strain were limited due to the current collector design (Fig. 1J). Furthermore, to the best of our knowledge, the effect of active mass loading and capacity on the mechanical properties of stretchable battery electrodes has yet to be investigated in detail.

Note that previous fluid batteries (including most existing non-fluid stretchable batteries) use unsustainable metal-based active materials (*7*). The use of these finite materials has a negative environmental impact, and many of the materials have been classified by the European Union as critical raw materials (*50*). Their production typically involves energy-intensive mining and extraction (*51*), and the ecotoxicity of liquid metals is debatable (*52*). To address the sustainability issue, batteries using redox-active organic materials, especially from biomass, are emerging as a sustainable alternative to their metal-based counterparts (*53*–*56*). In this report, we used modified lignin as a cathode active material. Lignin is one of the most abundant biopolymer on earth and is typically a waste product from the pulp industry and ethanol biorefinery (*57*). Approximately 100 million tons of lignin is extracted annually, with less than 5% being considered a value added commodity. The remaining are either discarded or burned for energy (*58*, *59*). Lignin contains quinone groups that can undergo an electrochemically induced reversible two-electron/two-proton redox reaction to permit energy storage (*60*–*64*). Repurposing a waste product into a value added commodity such a battery material would provide economic and sustainability merits. Therefore, it is important that future stretchable battery designs simultaneously address the mechanical, electrochemical, and sustainability considerations.

In this work, we demonstrate a stretchable battery design based on intrinsically deformable redox-active cathodic and anodic electrofluid electrodes that were incorporated into a full cell by a custom designed stretchable current collector and separator membrane (Fig. 1B). The key innovations are that the mass (volume) loading of the fluids and their resulting battery capacity do not influence the axial stiffness of the cell (Fig. 1E and fig. S1 for the corresponding battery fabrication and characterization) and that a sustainable conjugated polymer redox couple was used as active materials in the fluids. Figure 1F illustrates the fluid being extruded from a syringe with a toothpaste-like consistency (see the associated movie S1 and of a full cell cut in half with the fluid being squeezed out in movie S2). The molecular structures of the conjugated polymers used as redox-active particles are depicted in Fig. 1 (G to I). The design enables more active material mass loadings in the electrodes with higher battery capacities without a trade-off in their mechanical properties. This is an important stretchable battery parameter that has yet to be studied and is crucial for integration into next-generation wearables as discussed earlier. In comparison to metal-based redox-active fluid stretchable batteries, our fluids are based on sustainable redox-active organic materials, and our battery performance showed comparatively better electrochemical cyclic stability and mechanical robustness (see Fig. 1J and table S1). The battery could be stretched and twisted while operating a red light-emitting diode (LED) (Fig. 1K and movie S3). Furthermore, the axial stiffness remained relatively constant despite changing the topology and the volume of the fluids (Fig. 1, L and M, and fig. S2). The bending force of the rectangular fluid bags of different volumes was also relatively constant (fig. S2, H to L). The current collector consisted of a bilayer configuration of a nanographite (NG) and poly-styrene-ethylene-butylene-styrene (SEBS) conducting elastomer composite top buffer layer and a thin silver-nanowire (AgNW) mesh as the bottom layer embedded on a stretchable low permeability SEBS encapsulation to mitigate leakage and drying of the fluid. The membrane separator is made from a poly-styrene-isobutylene-styrene (SIBS) elastomer, and the porosity was induced using a solvent evaporation-induced phase separation method (*10*). The separator provided good proton conductivity while preventing crossover of the solid active materials in the respective cathodic and anodic fluids.

## RESULTS

### Redox-active electrofluid characterization

For the fluids, we used a model redox-active conjugated polymer system that was previously reported as solid-state electrodes by Crispin and coworkers. The wood-based biopolymer lignin (L) was modified and used as the cathode (*62*), and a conjugated polymer poly(1-amino-5-chloroanthraquinone) (PACA) as the anode (*65*). Both active materials were twined with a conducting polymer poly(3,4-ethylenedioxythiophene (PEDOT) (Fig. 1H) via in situ oxidative polymerization in the presence of the respective redox-active polymers to facilitate electrical transport within the particles (see experimental method in the Supplementary Materials). The active polymer composites will be referred to as PEDOT-lignin (PL) and PEDOT-PACA (PP). The molecular structures and electron-proton transfer redox reaction of lignin and PACA are shown in Fig. 1 (G and I, respectively).

**Fig. 2.**
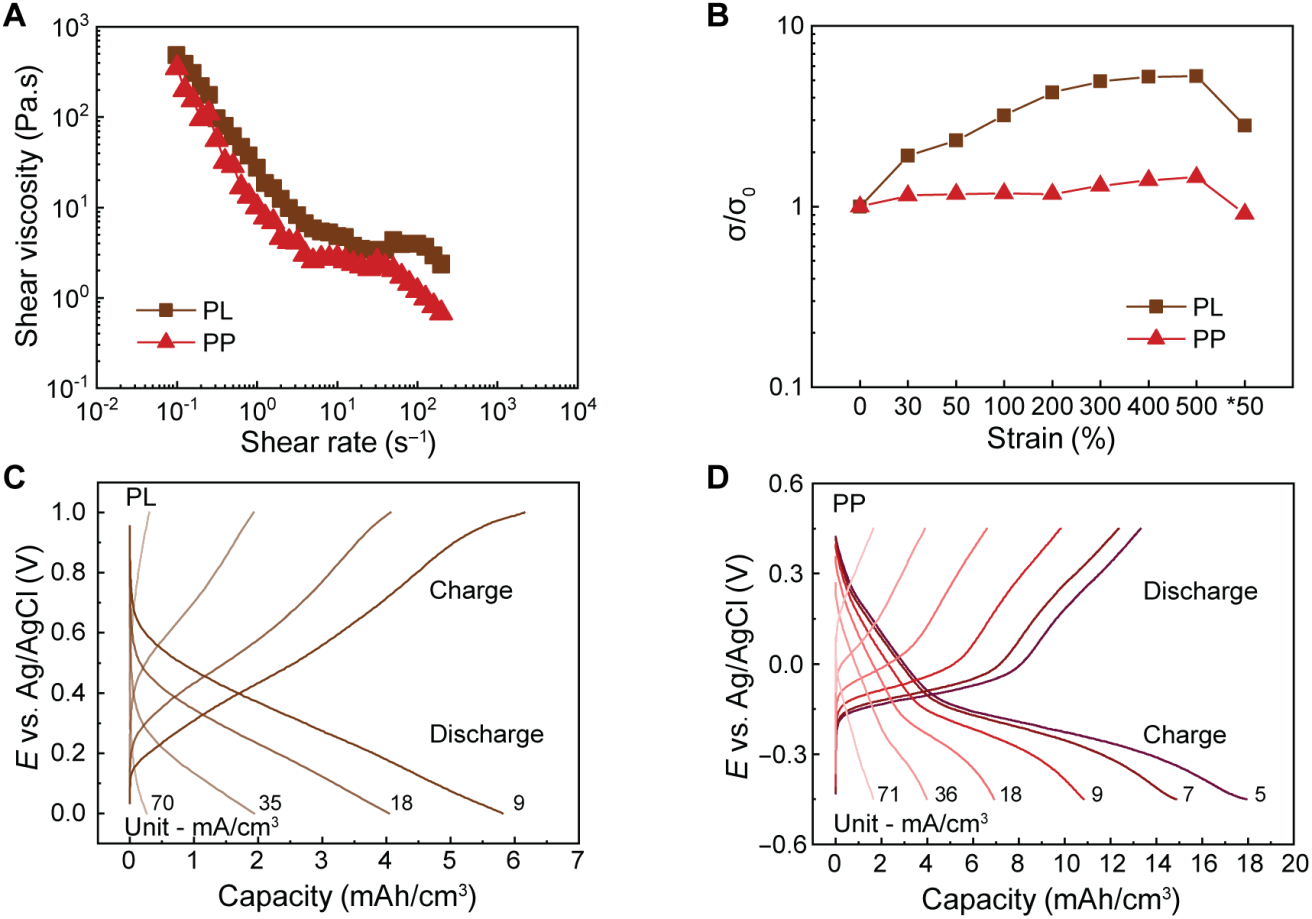
Redox-active electrofluid characterization. (**A**) Shear viscosity of the PL and PP fluids versus shear rate. (**B**) Normalized conductivity (σ/σ_0_) versus uniaxial tensile strain of the fluids. Half-cell GCD curves at different applied currents for (**C**) PL and (**D**) PP.

Fluidity was engineered into PL and PP by dispersing them separately with conductive carbon fillers in an electrolyte medium [0.1 M of HClO_4_ (aq)] at an approximate total weight concentration of 21 weight % (wt %) without any binder. The estimated particle size of PL and PP in the fluids was approximately 13 and 12 μm, respectively (fig. S3). The incorporation of carbon fillers was required to improve the electrical conductivity of the fluids (see fig. S3 for further description of the experimental method). Electrical characterization was performed by sandwiching the fluids between two electrode plates while applying a bias of different voltages over the fluid. The current was measured, and the resistance was extracted from the slope of the *E* versus *I* plot. The resistance of the fluids decreased by more than 90% from 11.4 to 0.83 kilohm(s) for PL and 12.5 to 0.49 kilohm(s) for PP upon the addition of the carbon fillers. The optimized fluids exhibited shear-tinning behavior, characteristic of non-Newtonian fluids, as indicated in the gradual decrease in viscosity with increasing shear rates (Fig. 2A). This behavior is similar to the semi-solid flowable slurries used in large-scale flow batteries. (*37*) Electrochemical impedance spectroscopy (EIS) was then used to extract the conductivity of the slurries under mechanical strain (Fig. 2B and see fig. S5, A and B for the configuration of the fluids contained within a SEBS casing and fig. S5, C and D for the corresponding bode plots). PL showed a fivefold increase in conductivity from 0.25 to 1.31 S/m as the applied strain on the fluid increased from 0 to 500%, while the conductivity for PP showed a smaller increase from 0.33 to 0.48 S/m. The conductivity of both fluids recovered toward its initial value when the strain was released. We speculate that the variation in electromechanical behavior could be due to differences in viscosities (Fig. 2A) or the nature of the polymers [e.g., polarity, particle size and dispersity (fig. S3)] and their interactions with the conductive carbon fillers. These factors likely influenced the kinetics of the system and any morphological changes as the fluid experience mechanical deformation, such as better alignment of particles leading to a well-connected percolation pathway for higher conductivity (*32*, *66*). The samples recovered back to approximately 50% of their original length due to the irreversible elongation of the SEBS encapsulation (fig. S5E).

To elucidate the energy storage capability of the fluids, half cells were fabricated to characterize their electrochemical properties (see schematic of the half cell in fig. S6A). Galvanostatic charge-discharge (GCD) curves of the fluids exhibited an increase in their volumetric capacity with decreasing applied currents, and a maximum attainable capacity of 5.92 and 13.5 mAh/cm^3^ was obtained for PL and PP at an applied current of 9 and 5 mA/cm^2^, respectively (Fig. 2, C and D). The capacity was normalized to the total volume of the fluid in the cell, including the electrolyte, active materials, and carbon fillers. The cyclic voltammetry (CV) curves (fig. S6, B and C) and gravimetric capacities (fig. S6F) are shown in the Supplementary Materials. The GCD curves of PP showed a clear plateau in the region of −0.2 to 0.05 V, which likely corresponds to the multistep 6H^+^/6e^−^ redox reaction of PACA (Fig. 1I), and the subsequent linear capacitive region of −0.05 to 0.4 V likely comes from both the carbon and PEDOT (Fig. 2D). For PL, the plateau region is not as obvious (Fig. 2C), and their CV data (fig. S6B) show broad redox peaks, which are typical of lignin-based electrodes, (*60*–*62*) including the CV curves for PP (fig. S6C). Both PL and PP show an improvement of their volumetric and gravimetric capacity with the addition of carbon fillers as summarized in fig. S6I. Our capacity values are comparable to previous reports from solid-state electrodes, and their energy storage process can be attributed to the capacitive contribution of PEDOT and the carbon fillers, as well as the faradaic proton-electron charge transfer reactions due to the quinone units in PL and PP, with PP having amino groups as additional secondary redox sites (*62*, *65*). Encouraged by the electromechanical and electrochemical properties of the conductive redox-active fluids, we proceeded with the design and fabrication of the stretchable current collector and separator membrane to demonstrate the stretchable redox fluid battery concept.

### Stretchable current collector and encapsulation

**Fig. 3.**
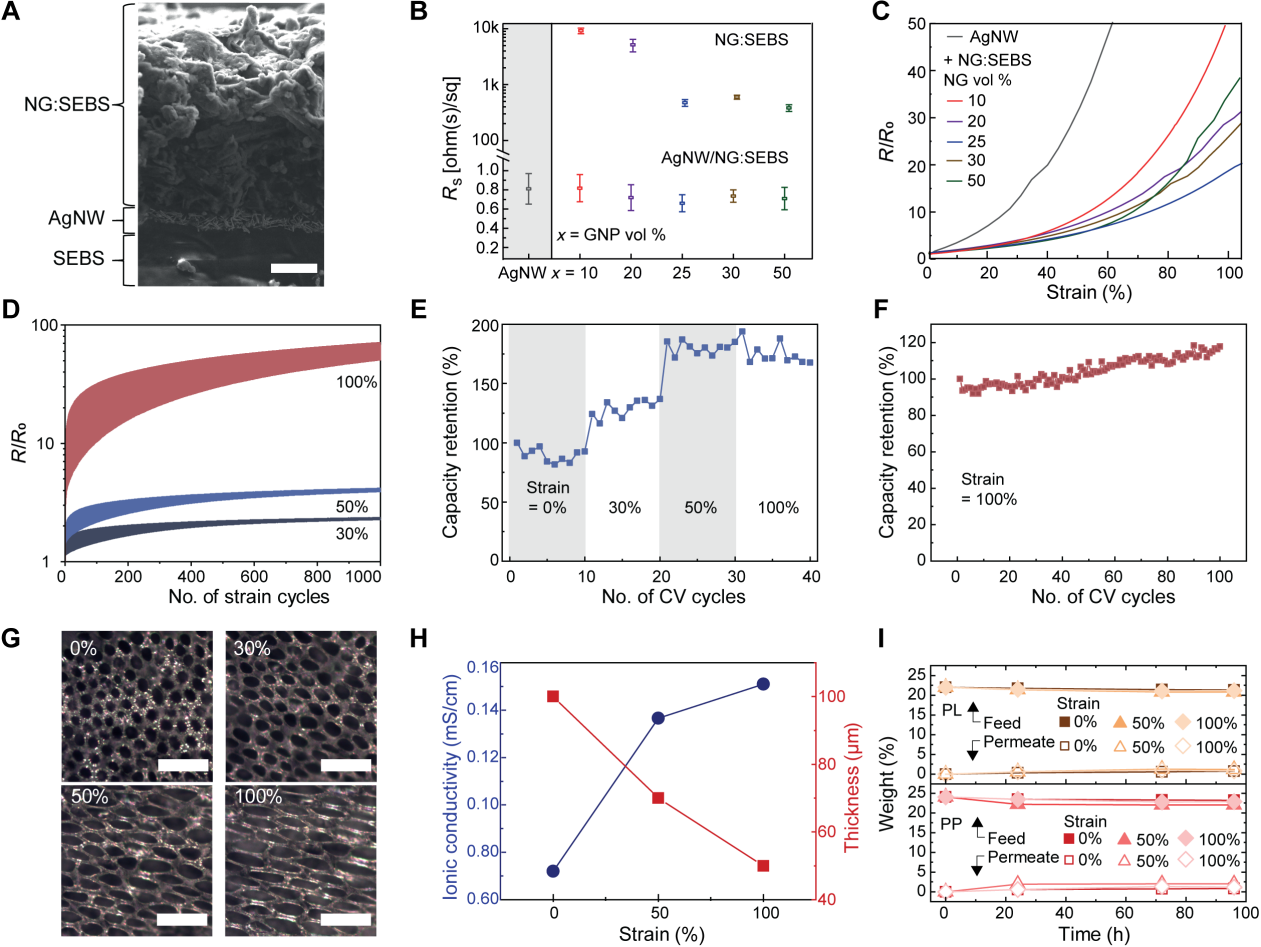
Stretchable current collector and separator membrane. (**A**) Scanning electron microscopy (SEM) cross section image of the current collector stack. Scale bar, 1 μm. (**B**) Initial sheet resistance of NG:SEBS layers of various loadings and AgNW-GNP:SEBS stacks. (**C**) Relative resistance under strain for AgNW-NG:SEBS stacks of various GNP loadings. (**D**) Strain cycling of the optimized current collector at different strains. (**E**) Capacity retention of the current collector in 0.1 M HClO_4_ (aq) electrolyte over 10 cycles at different strains (0, 30, 50, and 100%). (**F**) Capacity retention over 100 CV cycles at 100% strain. (**G**) Optical microscopy images of the SIBS separator membrane at different strains. Scale bars, 50 μm. (**H**) Ionic conductivity (left) and thickness change (right) of the membranes at different strains. (**I**) Membrane crossover of PL (top) and PP (bottom) fluid electrodes at 0, 50, and 100% strain over 96 hours.

An ideal stretchable current collector should have a low sheet resistance (*R*_s_) within the relevant strain range of the battery to ensure low ohmic losses while delivering current to the electrodes. In our design, a bilayer configuration was used for the stretchable current collector, with an optimized ≈ 324-nm-thick AgNW bottom layer embedded onto an 80-μm-thick SEBS encapsulation and a ≈ 4.13-μm NG:SEBS top buffer layer (Fig. 3A). AgNWs were selected as their high aspect ratio NW structure forms a mesh-like network that can induced high electrical conductivity and stretchability into the current collector (*67*). The NG:SEBS buffer layer protects the underlying AgNW mesh from corrosion and electrochemical oxidation due to the acidic electrolyte, and it improves the electromechanical performance of the current collector.

We first optimized the electromechanical properties of the respective conducting layers. NG:SEBS composites showed a relatively high *R*_s_ ranging from 384 to 9210 ohm/sq as the vol % of NG decreased from 50 to 10 vol % (Fig. 3B), which is expected for carbon-based conducive fillers (*10*, *24*, *25*). The AgNWs layer was optimized to minimize the amount of unsustainable metal components without sacrificing its conductivity (fig. S7) (*51*). When the optimized underlying AgNW layer was introduced into the current collector configuration, the *R*_s_ decreased significantly to 0.74 ± 0.06 Ω/□, and minimal variations were observed between the different vol % of NG. The bilayer current collectors with different vol % of NG showed different electromechanical behavior (Fig. 3C). In a single-layer configuration of AgNW on SEBS, the normalized resistance (*R*/*R*_0_) increased to more than 20 times its original value at 50% strain, but in a bilayer configuration, the *R*/*R*_0_ remained below 5. At 25 vol % of NG, an optimum electromechanical performance was observed. This behavior is attributed to the formation of a secondary electron percolation pathway that bridges low-conductivity regions within the AgNW network (*26*). For NG loadings above 25 vol %, the electromechanical properties of the current collector deteriorated slightly, probably due to a lower amount of elastomer in the composite. Using the optimized condition, cyclic tensile tests of the current collectors were performed on the bilayer current collector (Fig. 3D). It exhibited a stable change in resistance at 30 and 50% strain cycling with an *R*/*R*_0_ below 2. At 100% strain cycling, the *R*/*R*_0_ increased gradually to above 50 over 1000 strain cycles. This is likely due to the plastic deformation of the SEBS elastomer under prolonged stress above its elastic regime (fig. S5E).

Electrochemical experiments were performed to investigate the capability of the front buffer layer and the back SEBS encapsulation in protecting the AgNW layer against degradation under bias. Three-electrode CV measurements of electrodes consisting of a single AgNW layer embedded on SEBS that was exposed to the acidic electrolyte solution were performed. The CV showed a rapid electrochemical corrosion of the AgNWs that was visible as an irreversible oxidation peak due to the formation of Ag^+^ during the first CV cycle (fig. S8A) (*68*). The formation of salt particles was observed in the SEM images of corroded AgNWs, indicating a disruption in the conducting network of the layer (fig. S8B). In a bilayer configuration, a reversible quasi-rectangular voltammogram was observed (fig. S8C), and to demonstrate the electrochemical stability of the current collector, 10 CV scans were performed in their stretched state at 0, 30, 50, and 100% strain. The capacitance remained relatively stable over the 10 cycles at each strain level, and it increased to approximately 125 and 180% of its initial value at 30 and 50% strain, respectively (Fig. 3E). At 100% strain, a slight decrease in capacitance was observed to approximately 175%, likely due to the increase in resistance of the current collector as shown in Fig. 3D.

We then performed 100 CV cycles on the stretched current collector, and the capacitance remained relatively stable (Fig. 3F and see fig. S8G for the CV curves). Scanning electron microscopy (SEM) images of the bilayer current collector at 100% strain did not show any exposed underlying AgNWs (fig. S8H), indicating the ability of the NG:SEBS composite to function as a robust electrochemical and mechanical buffer layer. Reversible redox peaks were observed in the CV curves of the current collector with the buffer layer (fig. S8, C to G). They likely correspond the surface redox reaction of Ag(0) to Ag(I) on the AgNW, and it seems like the buffer layer suppresses the dissolution of the Ag(I) ions into the electrolyte (*69*), and even under 100% strain, the capacitance of the electrode remains stable (Fig. 3, E and F). Notably, diffusion of the electrolyte from the back of the current collector via the SEBS encapsulation protected the AgNWs (Fig. 3A). Considering that the electrodes are fluids, the role of the encapsulation is critical in preventing the electrolyte and fluids from drying out. To evaluate this property, a SEBS bag containing a liquid of a dye molecule dissolved in the electrolyte was prepared (fig. S9A). SEBS glue was used to ensure proper sealing of the SEBS layers, and the bag was immersed in an electrolyte solution (fig. S9B). The absorption spectra of the solution were taken over several weeks before and after stretching, and no obvious indication of the dye molecule leaking out the bag was observed (fig. S9, C and D). This suggests that the gluing method ensures good adhesion between the different layers, and the low permeability of the SEBS layers under strain can prevent leakage of the electrolyte.

### Stretchable porous separator membrane

The primary function of the stretchable separator membrane is to electrically isolate the electrodes from each other while allowing ion transport. In this case, mainly protons are transported between the cathode and the anode to facilitate redox reactions at the respective electrodes. The membrane must also mitigate any crossover of the active particles in the fluids under mechanical strain to avoid degradation of the battery capacity over time. A solvent evaporation-induced phase separation method was used to fabricate the porous SIBS separator membrane (*10*). Briefly, SIBS was dissolved in a binary solvent system containing toluene and dimethyl sulfoxide (DMSO), and upon casting, because of their immiscibility and different degrees of volatility, phase separation occurs with toluene evaporating at a faster rate that starts from the solution/air interface (top side) that moves downward to the bottom of the solution. This resulted in a in a hierarchical porous structure with pore sizes between 8 and 11 μm at 0% strain at the top side of the membrane (Fig. 3G) and pores around 400 μm in diameter, with the smaller pores within it, on the bottom side (fig. S10). A similar observation was also seen in previous work using the same method (*10*). The ionic conductivity of the membranes (Fig. 3H) was measured as a function of uniaxial tensile strain while considering the change in thickness using a four-point probe EIS technique in an H cell (see fig. S11A for the corresponding bode plots and for the H cell setup). For a detailed description, see the Materials and Methods. As the membrane was stretched from 0 to 100% strain, an increase in ionic conductivity from 0.071 to 0.15 mS/m was observed. This can be attributed to the formation of larger pores (Fig. 3G) as the membrane was stretched which reduces ionic transport distance (Fig. 3H ). To evaluate the crossover of the active materials through the membrane under strain, permeation tests were conducted over a 96-hour period at 50% strain [Fig. 3I and see fig. S11, C to L for the concentration calibration curves of the PP and PL fluids and their corresponding ultraviolet-visible (UV-vis) absorption spectra]. The concentration of the permeates for both fluids remained below 1% after 96 hours in the relaxed state. At 50 and 100% strain, only a minimal increase in concentration to 3% was observed, which could be due to a slightly larger pore size of 10 to 30 μm (Fig. 2G). The hierarchical porous network of the membrane likely introduces higher tortuosity for particle movement that minimizes crossover despite an increase in pore size (fig. S10).

### Stretchable full cell characterization

**Fig. 4.**
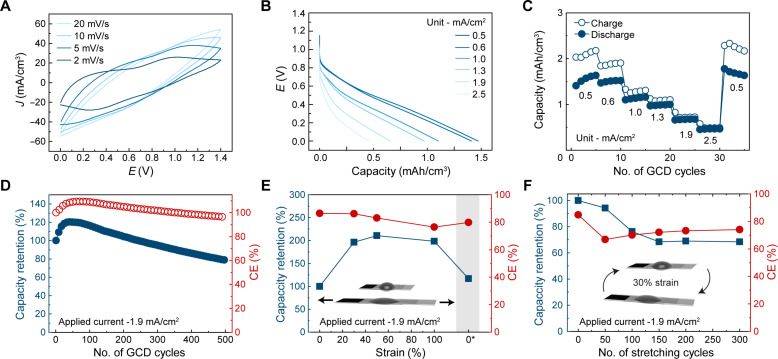
Full cell characterization. (**A**) CV curves at various scan rates. (**B**) Rate capability of the full cell showing the representative discharge capacity curves taken from the first GCD cycle and (**C**) the GCD capacities over five cycles each at different current densities. Normalized capacity retention taken from the (**D**) GCD cycling of the full cell at rate of 1.9 mA/cm^2^, (**E**) at various static strains (0, 30, 50, and 100% and 0* indicates the release of the strain from the cell), and (**F**) under constant cyclic stretching at 30% strain over 300 cycles.

The full cell was assembled with a total (cathodic and anodic) fluid volume of 0.092 cm^3^ using the optimized stretchable battery components (see fig. S1D for a schematic of the fabrication process). CV curves at different scan rates of 2, 5, 10, and 20 mV/s from 0 to 1.4 V were performed (Fig. 4A). The voltammograms exhibited broad reduction and oxidation peaks between 0.0 to 0.6 V and 0.6 to 1.4 V, respectively, which corresponds to the proton-electron charge transfer mechanism and the capacitive contributions of PEDOT and the carbon fillers as discussed earlier. Figure 4B shows the galvanostatic discharge curves taken from the first cycle, and the rate capability at different current densities (0.5, 0.6, 1, 1.3, 1.9, and 2.5 mA/cm^2^) is reflected in Fig. 4E. The corresponding gravimetric capacities are shown in fig. S12A. An initial increase in capacity was observed from the first to the fifth cycle from 1.49 to 1.76 mAh/cm^3^ at a rate of 0.5 mA/cm^2^. The coulombic efficiency (CE) at this rate remained approximately at 80%, but as the applied current gradually increases to 2.5 mA/cm^2^, the CE gradually improves close to 100%, and the capacity decreases to 0.46 mAh/cm^3^. At low current densities, the electrodes are more prone to water splitting parasitic side reactions and the electrochemical degradation of the quinone groups in the conjugated polymers, resulting in a lower CE. This is a separate yet critical issue that needs to be addressed for redox-active organic materials (*70*). A voltage drop from ≈0.9 to 0.7 V was also observed as the applied current increases and is attributed to ohmic losses occurring at higher current rates (*71*). Nevertheless, the cell showed good rate capability with stable capacity retention at each applied current and recoverability when returning to slower rates of 0.5 mA/cm^2^. Furthermore, GCD cyclic performance of the cell showed a good capacity retention of 85.2% over 500 cycles at 1.9 mA/cm^2^. The slight reduction in the cell capacity is also linked to the instabilities of the redox-active quinone groups in lignin (*70*).

Next, the mechanical performance of the cell was evaluated. The cell was first charged and discharged at its initial state of 0% strain and subsequently stretched to 30, 50, and 100% strain, and a GCD cycle was measured in the respective stretched states (Fig. 4E and see fig. S12A for their corresponding GCD curves). An initial increase in capacity of *C*/*C*_0_ ≈ 1.9 was observed when the cell was stretched to 30% strain, which increased to *C*/*C*_0_ ≈ 2 at 50 and 100% strain, after which the cell recovered close to its original capacity *C*/*C*_0_ ≈ 1.2 as the strain was released. It is likely that the capacity increases in the stretched state due to an increase in ionic conductivity in the membrane (Fig. 3H), and a decrease in overall thickness of the cell reduces both ionic and electrical resistances across the cell. The current collectors may also have affected the performance because a higher surface area in the stretched state could have facilitated better interfacial contact with the fluid, as observed in the increase in capacitance as the current collector was stretched (Fig. 3E). Encouraged by the mechanical performance of the battery, we evaluated its robustness by stretching the cell for 300 cycles at 30% strain, and a GCD cycle was measured in its initial state of 0% strain at every 50 cycle intervals. The results showed a 70% capacity retention after 150 cycles at 30% strain, which remained stable up to 300 stretching cycles (Fig. 4F and see fig. S12B for their corresponding GCD cycles). The internal resistance across the cell increased slightly from 33.5 to 50 ohm(s) at its initial state and after 300 strain cycles as shown from the EIS measurements (fig. S12C). This can be attributed to the slight increase in sheet resistance of the current collector under strain cycling as shown in Fig. 3D. Bearing in mind that the biomechanical environment of on-skin and implanted wearable devices experience mechanical deformations of less than 30% strain, the mechanical performance of the developed cell would constitute a suitable power supply unit for these applications (*72*). The mechanical cycling test indicates that the encapsulation and adhesion of the various battery layers prevent leakage under mechanical strain (Fig. 4, E and F). To provide further evidence, the full cell was immersed in a fixed volume of water at pH 7, and the measured pH remained relatively stable over 6 days (fig. S12D). The cell was then stretched for 100 cycles at 30% strain and was reimmersed in the water, and the measured pH did not change significantly over a period of 7 days (fig. S12E). This demonstrates the robustness of the encapsulation and the gluing method (fig. S9) to adhere the battery components together and to protect the internal fluids from leaking out even after applying mechanical strain to the full cell.

## DISCUSSION

In conclusion, an electrode design concept was developed to create intrinsically stretchable battery electrodes by tuning the viscosity of fluids instead of the Young’s modulus of solids. The decoupling of the mechanical and the electrochemical function of the electrode resulted in a stretchable battery where its capacity and active material loading are independent from the mechanical compliancy (axial stiffness) of the cell (Fig. 1E). This is an important characteristic that has yet to be demonstrated by existing stretchable battery designs (*7*). Overall, the redox-active electrofluids showed excellent conductivity retention and reversibility when deformed axially up to 500% strain. A customized designed stretchable current collector and separator membrane was essential in ensuring the overall mechanical robustness of the cell, which showed stable capacity retention and reversibility up to 100% strain and after 300 stretching cycles at 30% strain. The full cell showed good rate capability and a capacity retention of 85% after 500 GCD cycling. The low permeability of the SEBS elastomer encapsulation was also key in avoiding leakage of the fluids under strain. Future designs should aim at replacing the acidic electrolyte with a safer pH neutral and biocompatible equivalent, considering its application for wearables (*73*).

While the mechanical stiffness remains independent of thickness, the capacity only doubles when the fluid electrode volume increases by approximately 4 times (Fig. 1E). This is likely due to an increase in series resistance across the cell, which is also indicated by the rate dependency in capacity (Fig. 4C). To mitigate this effect, the rate capabilities of the redox active electrofluids can be improved in future work by optimizing particle concentration and balancing the fraction between the conductive and redox-active particles. Different mixing methods (e.g., ball milling and shear mixing) can increase the interfacial contact between the particles leading to an improvement in the access to the active particles, which ultimately enhances capacity with increasing fluid volume. The volumetric energy and power densities of the full cell are reflected in a Ragone plot in fig. S13A and their gravimetric capacities in fig. S13B. Improvements using higher energy density battery chemistries, such as lignin and Zn-ion redox couples, can potentially increase the cell voltage up to 1.3 V with excellent capacity retention (≈80%) over 8000 GCD cycles and comparatively higher specific energy and power densities (*74*). Future work should also involve battery chemistries that use sustainable materials with higher energy densities (*7*). As compared to the handful of reports (Fig. 1G and table S1) that uses fluids as active electrodes in stretchable batteries, our fluid concept enables better mechanical robustness and uses a sustainable conjugated polymer redox couple system (particularly the lignin cathode). This is in stark contrast to the unsustainable metal-based active materials that generally suffer from electrochemical instabilities and are detrimental to the environment when considering their life cycle (*50*–*52*). Specifically, liquid metal gallium has the risk of converting into a solid oxide phase during battery operation, losing its fluidic nature and thus affecting its electrochemical performance and mechanical compliance. Our redox-active electrofluid concept opens the door for future developments of sustainable stretchable battery designs with unique properties such as thixotropic or rheopectic fluids in which the materials can provide solid characteristics at rest and fluidic properties under (mild) stress or vice versa. By exploiting the unique properties of fluids, it could enable batteries with an embedded dual (sensing) function that alters its discharge current upon activation of an external stimuli (*28*). In addition, the fluids potentially simplifies electrode manufacturing by removing the need for slurry casting and drying of the electrodes. With the highly deformable nature of the fluids, form factor–free battery configurations can be achieved that are highly suited for integration in next-generation wearable devices (*35*).

## MATERIALS AND METHODS

### Synthesis of redox-active polymers

PL and PP were synthesized according to previous reports (*62*, *65*).

### Fluid preparation

To prepare the PL fluids, 1 g of a suspension of PL in deionized (DI) water (16 wt %) was mixed with 80 mg of activated carbon (AC) (YP-50F, Kuraray Co. Ltd.) and 22 mg of carbon black (CB) (ENSACO 360 G from IMERYS) at weight PL:AC:CB ratios of 2:1:0.28. An electrolyte solution of HClO_4_ (aq) (perchloric acid, Sigma-Aldrich, ACS reagent, 70%) was added to reach a final concentration of 0.1 M. A planetary centrifugal mixer (ARE-250, Thinky) was used to ensure uniform dispersion of the solid components in the electrolyte. Four mixing cycles of 1 min at 2000 rpm followed by 30 s of degassing at 2200 rpm resulting in a final concentration of approximately 21 wt % of dry material. A similar procedure was used to prepare the PP fluids. Briefly, they were prepared by dispersing 1 g of a suspension of PP in DI water (20 wt %) with 200 mg of AC and 40 mg of CB and 0.1 M HClO_4_ (aq) at weight PP:AC:CB ratios of 1:1:0.2., resulting in a final concentration of approximately 24 wt % of dry material. The fluids were used for the subsequent rheology, electromechanical, and electrochemical (half cell and full) characterizations. For carbon (activated carbon + carbon black) only electrofluids, a total concentration of 6.4 and 10.9 wt % were used, respectively, for the GCD experiments in fig. S6.

### Fluid rheology

The fluids were prepared without electrolytes and preshearing, and their viscosity was measured at room temperature (25°C) using DI water at the same concentration. The measurements were conducted with parallel plates (25-mm-diameter upper plate and 40-mm-diameter lower plate) at shear rates ranging from 0.1 to 200 s^−1^ using a Malvern rheometer (Kernel version 5.00).

### Particle size analysis

Particle size analysis was performed using a particle size analyzer (Mastersizer 3000, Malvern Panalytical) by diluting the fluids (without the electrolyte) in excess DI water.

### Device fabrication and electromechanical characterization of fluids

Two gold-coated copper pads were placed on a SEBS (TUFTEC H1052, Asahi Kasei Elastomers) substrate, and a 1-mm-thick SEBS gasket was glued on to the substrate using SEBS glue. The cavity was filled with the respective fluids and encapsulated with an 80-μm-thick SEBS films using SEBS glue. Excess solvent from the glue was evaporated before clamping the device on to a custom-made computer-controlled motorized linear stretching stage (LSQ300A-EO1, Zaber). A two-electrode sandwich structure was used to measure the out-of-plane resistance of the fluids. The impedance was measured using a Gamry 1001E potentiostat across a frequency range of 0.1 to 10 kHz with an AC voltage of 10 mV, and the conductivity was deduced from the measured impedance of the system in the resistive regime and calculated using following formula: *s* = *h*/(*R*_s_ − *R*_0_) *A*, where *s* is the conductivity and *h* and *A* are the thickness and the area fluid, respectively. The change in geometry was assumed on the basis of an incompressible material system with a Poisson’s ratio (𝜈) of 0.5.

### Half-cell characterization

A three-electrode setup was used with Pt coil as the counter and Ag/AgCl (3 M KCl) as the reference electrode. The working electrode consisted of an Au/Cr current collector evaporated on glass with the fluid confined in a cavity of a fixed volume. A porous polyvinylidene difluoride membrane (pore size of 200 nm) was attached over the cavity to prevent leakage of the slurry into 0.1 M HClO_4_(aq) of the electrolyte solution of the flooded cell. GCD characteristics were measured using a Gamry 1001E potentiostat.

### Current collector—AgNW Filtration and transfer on SEBS

A filtration transfer method was used to prepare the current collectors. First, a hydrophilic cellulose nitrate membranes (0.22-μm pore size, Millipore) was soaked in DI water and then mounted onto a filtration setup. An 80-μm-thick mask made from SEBS (TUFTEC H1052) was laser-patterned (MetaQuip Laser engraving machine, FMHUV3W) and then placed on the membrane, and vacuum was applied. Ag nanowires (NW) (60-nm diameter, 10-μm long, 10 mg/ml in isopropanol, NovaWire-Ag-A60SL) solution were diluted in 20 ml of DI water, vortexed for 30 s, and filtered through the membrane to obtain a patterned AgNW current collector. The membrane was then dried using an N_2_ gun. The optimized surface coverage of the AgNW pattern was 0.03925 mg/cm^2^. The SEBS encapsulation was prepared by dissolving in toluene (Fisher Chemical) (400 mg/ml), spin-coated on a clean glass substrate at 500 rpm for 40 s, and left on hot plate at 70°C for 90 s. The patterned AgNW on the membrane was carefully placed over the “wet” SEBS substrate, and pressure was applied on it with a 1 kg in weight at 70°C for at least 2 min to ensure that the AgNW was embedded into the SEBS elastomer. The membrane was then dissolved in acetone (VWR, 99%), resulting in SEBS elastomer substrate embedded with the AgNW pattern.

### NG:SEBS buffer layer deposition over AgNWs

NG (Nanostructured Graphite-250, Graphene Supermarket) and SEBS (TUFTEC H1052, Asahi Kasei Elastomers) were mixed at various volume ratios in toluene (100 mg/ml). The solution was shear mixed (T10 basic ULTRA-TURRAX) at speed level 6 for 15 min and then left to stir overnight at room temperature (R.T.). The solution was then doctor-blade using a 100-μm-thick plastic mask over the AgNW and dried at R.T. for 1 hour to evaporate excess toluene. An electrical connection extension was formed by doctor blade coating a stretchable conductor ink using a 100-μm-thick plastic mask onto the end of the current collector, enabling easier electrical connections and handling. The ink was made by mixing SIBS in toluene (100 mg/ml) with Ag flakes powder (47MR-11F, Inframat Advanced Materials) to achieve a final concentration of 80% (v/v) Ag flakes in SIBS. A thin layer of 20-μm SEBS gasket with a laser-patterned circular opening (diameter = 1 cm) was glued to the current collector to avoid any electrical connection between the cathodes and anodes. The electromechanical properties of the assembled current collectors were measured with custom-made computer-controlled motorized linear stretching stage (LSQ300A-EO1, Zaber) with an integrated digital multimeter data acquisition system (Keithley 2701 Ethernet) connected to four-point gold-coated probes. The electrochemical stability experiments were conducted using a three-electrode setup in a beaker containing 0.1 M HClO_4_(aq), with Pt-coil as the counter electrode, Ag/AgCl as the reference electrode, and current collector with and without the buffer layer as the working electrode. All electrochemical measurements were performed using a Gamry 1001E potentiostat. Morphological characterization of the current collectors was performed using scanning electron microscopy (Sigma 500 Gemini, ZEISS).

### Membrane preparation and characterization

The fabrication of the stretchable separator was adapted from a previous report (*10*). Briefly, a DMSO (99.5 wt %; Sigma-Aldrich, ReagentPlus) was added dropwise into a solution of SIBS (103 T-UL, SIBSTAR, KANEKA Corporation) in toluene to obtain a final volume ratio of 1:10 DMSO/toluene and left to stir for at least 2 hours before drop-casting into a 100-μm-thick SEBS mould that was laser-patterned with a rectangular template of 2 cm by 4 cm on a glass substrate. The sample was left overnight to enable the solvents to evaporate to obtain the membrane. The membranes were then soaked in 0.1 M HClO_4_(aq) overnight before characterization. Before placing the membrane in a H-cell setup (fig. S11), the membrane was placed in between two SEBS gaskets with a circular opening of 0.785 cm^2^. The ionic conductivity of the membrane in its initial and uniaxially stretched state was obtained by measuring the impedance using a four-point method across the electrolyte with and without the membrane in an H-cell setup. For ionic conductivity under strain, the membrane was uniaxially stretched at the respective strains (50 and 100%) before sandwiching in between the gaskets, and the area of the opening remained the same at 0.785 cm^2^. The thickness was measured accordingly in their stretched state using a micrometer screw gauge. The impedance was measured across a frequency range of 0.1 to 10 kHz with an AC voltage of 10 mV using two reference electrodes (Ag/AgCl in 3 M KCl) to detect the local potential drop and two coiled platinum wire electrodes to apply the AC current. Ionic conductivity was deduced from the measured impedance of the system with (*R*_s_) and without (*R*_0_) the membrane using following formula: *s* = *h*/(*R*_s_ − *R*_0_) *A*, where *h* and *A* are the thickness and the area of the separator, respectively. A Sigma 500 Gemini (ZEISS) used for obtaining SEM images of the membrane. Optical microscope images of the membrane in initial and uniaxially stretched states were obtained using a Nikon Optiphot 150 inspection microscope in bright field mode.

### Membrane crossover

A dummy cell was first fabricated. It consisted of a 1-mm-thick SEBS gasket with a 1-cm-diameter cavity that was glued onto an 80-μm SEBS substrate. The cavity was then filled with the fluid, the separator membrane was placed over it, and SEBS glue was applied along the sides to ensure that permeation occurs only through the separator. The dummy cell was immersed in beaker containing 0.1 M HClO_4_(aq) in its initial and stretched state of 50% strain and 100% strain. UV-vis measurements were conducted at various intervals using a spectrometer (Lambda 900 UV/VIS/NIR Spectrometer, PerkinElmer). A similar experiment was conducted to test the permeability of the SEBS encapsulation.

### Encapsulation leakage test

To test the leakage, a SEBS bag containing a sealed dye solution of 0.01 M Alizarin red sulfonate (Sigma-Aldrich) in 0.1 M HClO_4_ (aq) was immersed in a beaker containing 0.1 M HClO_4_(aq) for 100 days. The absorption spectrum of the solution in the beaker was measured after 72, 144, and 192 hours. The bag was then stretched at 1 mm/s to 50% strain over 100 cycles using the linear stretching setup, and the concentration measurements were taken after 24 and 336 hours. To test for leakage in the full cell, a fabricated cell was immersed in 80 ml of water for 7 days, during which the water’s pH was measured daily using a pH meter (FiveEasy Plus, Mettler Toledo). For the stretching test, the cell was uniaxially stretched reversibly 100 times at 1 mm/s to 30% strain and then immersed in 80 ml of water for 7 days, with the pH measured regularly.

### Fluid topology test

Silicone molds (Dragon Skin 10 Slow, Smooth-On) with different cavity shapes and volumes were first fabricated. An 80-μm SEBS substrate was then gently placed into the cavity and pressed to remove air. The substrate then conforms into the shape of the cavity. The cavity was subsequently filled with PL. SEBS glue (200 mg/ml) was applied around the cavity using a cleanroom swab, and another 80-μm SEBS film was placed on top and pressed around the edges to ensure adhesion and to seal the fluids by the SEBS casings. The bag was then removed from the molds and set aside at room temperature to evaporate the toluene solvent for at least 2 hours before mechanical characterizations. A custom-made stress-strain setup, including a motorized linear stage (X-LSQ300A-E01, Zaber) and a force gauge (M5–2, Mark-10), was used for mechanical characterizations at a strain rate of 30% of the length of the sample per minute. The length of the sample is taken from the distance between the clamping points. For the bending test, the samples were displaced inward by 50% of the original distance of the clamping point to induce a bend in the sample structure.

### Full cell assembly and characterization

A schematic of the fabrication process is illustrated in fig. S1. Silicone molds (Dragon skin 10 Slow, Smooth-On) with different cavity volumes were first fabricated. A current collector was then gently placed in the cavity to take its form and filled with the respective cathodic and anodic fluids. SEBS glue (200 mg/ml) was applied around the cavity using a cleanroom swab. A SEBS (thickness of 1 mm) gasket was used to test the electrochemical properties of the full cell, while for the electrochemical-mechanical properties, the membrane was placed directly over the fluids and gently pressed around the edges to ensure adhesion and sealing of the fluids in the cavity formed by the current collector. The process was repeated for the other electrode but without the membrane. Both half cells were then sandwiched together using SEBS glue to obtain the full cell. The cell was then removed from the molds and set aside at R.T. to evaporate the toluene solvent for at least 2 hours before characterization. All electrochemical measurements (CV, GCD, and EIS) were performed using a Gamry 1001E potentiostat. For the mechanical testing of the battery performance, the cell was clamped on to a motorized linear stage (X-LSQ300A-E01, Zaber) and stretched at a rate of 5 mm/min, and the cell was connected to a Gamry 1001E potentiostat. For the mechanical properties of the cell with different fluid volume loading, a force gauge (M5-2, Mark-10) was used, and the cells were stretched at a rate of 6 mm/min. For the demonstrator, three cells were connected in series. To ensure a robust electrical connection, copper wires (⌀ = 100 μm) were directly soldered onto the stretchable conductor ends of the cells, which were then used to power an LED (SML-P11DTT86R).
