# Supplementary material for: Make it flow from solid to liquid: Redox-active electrofluids for intrinsically stretchable batteries
Source: Sci Adv. 2025 Apr 11;11(15):eadr9010. doi: 10.1126/sciadv.adr9010 (PMC11988450; doi:10.1126/sciadv.adr9010)
Supplement: Supplementary file 1 — Table S1 Figs. S1 to S13 Legends for movies S1 to S3 [file sciadv.adr9010_sm.pdf]

Science Advances
AAAS

Supplementary Materials for

Make it flow from solid to liquid: Redox-active electrofluids for intrinsically stretchable batteries

Mohsen Mohammadi *et al.*

Corresponding author: Saeed Mardi, saeed.mardi@kemi.uu.se; Klas Tybrandt, klas.tybrandt@liu.se; Aiman Rahmanudin, aiman.rahmanudin@liu.se

Sci. Adv. **11**, eadr9010 (2025)
DOI: 10.1126/sciadv.adr9010

The PDF file includes:

Table S1
Figs. S1 to S13
Legends for movies S1 to S3

Other Supplementary Material for this manuscript includes the following:

Movies S1 to S3

Table S1. Comparison of the electrochemical performance of reported fluid-based stretchable batteries. C/C_0 is the normalised capacity.

Active Materials	Battery design	[V]	Stretching Performance		GCD Cycle Stability	Areal Capacity [mAh/cm^2]*	Ref
			Max Strain	Reversible strain			
**Fluid-based anode and cathode**							
PP‖PL	Fluid-based electrode using redox-active polymers with an intrinsically stretchable current collector.	0.9	$C/C_0 \approx 2.0$ @ 100 % strain	$C/C_0 \approx 1.2$ after stretching to 100 % strain and ≈ 70 % retention for 300 cycles @ 30 % strain	107 % after 100 cycles @1.9 mA/cm^2	0.26 @ 0.5 mA/cm^2, Volumetric capacity equivalent 1.76 mAh/cm^3 @ 0.5 mA/cm^3	*This work
*Considering that fluids are used as a comparison; areal capacity does not reflect the actual capacity of the battery cell. Volumetric capacity considers the geometry of the whole electrode and should be a better metric of comparison. Only the areal capacity is shown since the comparison with other reports did not calculate their volumetric capacity or the geometry of their cell.							
Zn‖MnO_2	Fluid-based electrode and a carbon slurry current collector. Aqueous hydrogel electrolyte.	1.5	$C/C_0 \approx 0.49$ @ 50 % strain	Short circuit current $I/I_0 \approx$ 0.4 for 2 cycles @ 100 % strain	Not reported	3.5 @ 0.2 mA/cm^2	47
EGaIn‖MnO_2	New pairing of fluid-based active electrodes with Carbon-grease slurry and Cu film current collector. Aqueous hydrogel electrolyte.	1.4	$C/C_0 \approx 0.98$ @ 100 % strain	Stable @ 100 % strain for 5 cycles	≈ 99 % after 100 cycles @0.4 mA/cm^2	3.8 @ 0.2 mA/cm^2	46
Zn‖MnO_2	Fluid-based electrode and wavy thin film metal current collectors. Aqueous hydrogel electrolyte.	1.5	Internal resistance (Ω), $R/R_0 \approx$ 0.84 @ 40 % strain	Stable discharge @ 20% strain over ≈ 150 mins	31 % after 100 cycles	16.8 @ 10 mA	48
Zn‖MnO_2	Fluid-based electrode and a conductive textile mesh current collector. Aqueous hydrogel electrolyte.	1.5	$C/C_0 \approx 1$ @ 75 % strain	50 % capacity retention for 700 cycles @ 25 % strain	45 % after 22 cycles	1.1 @ 1mA	49
**Fluid-based Anode or Cathode only***							
V_2O_5@C// EGaIn@Zn	Fiber-based.	0.8	$C/C_0 \approx 1.0$ @ 50 % strain	83 % @ 50 % for 300 cycles	92.5 % after 600 cycles	168 mAh /cm^3 @ 2.0 mA/cm^3 , (Areal capacity not reported)	5
Pt-C// EGaIn	Fiber-based Metal air battery.	1.5	Discharge current	Not reported	98.9 % after 500 cycles	214.8 mAh/g[1] (areal capacity not reported)	6
MoO_3// Mg	Fluid based Cathode and kirigami Mg film as anode.	1.7	$C/C_0 \approx 1.5$ @ 20 % strain	Stable @ 20 % strain for 900 cycles	Non-rechargeable	4.7 mAh @ 45μAh/cm^2 (Areal capacity not reported)	7

Ag_2O//Ga	3D printing Active material, Fluid Ga-anode and composite cathode.	1.7	$C/C_0 \approx 2.4$ @ 130 % strain	Not reported	100 cycles at 0.4 mA cm^2	8.15	8

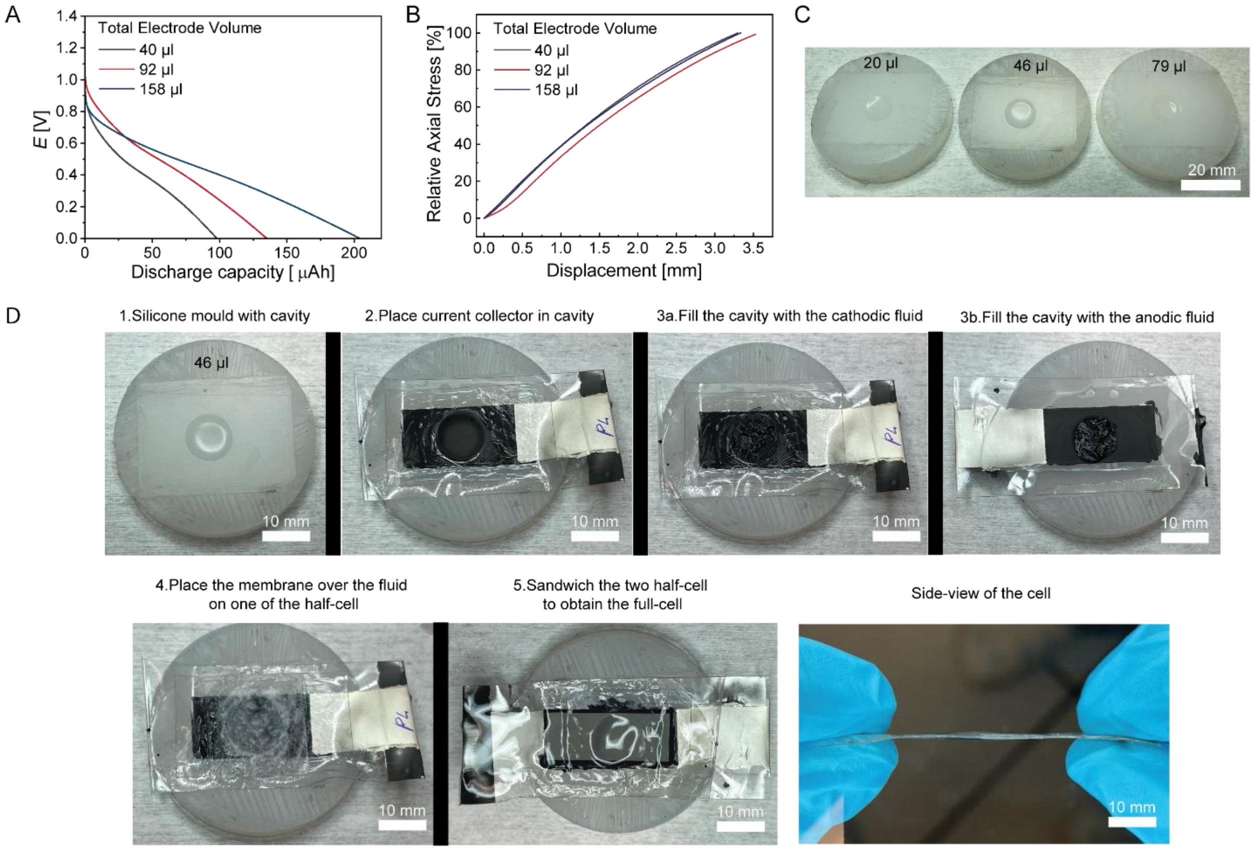

Figure S1. Battery characterization of the stretchable fluid cells with different volume loadings of the cathodic and anodic fluids. A) Galvanostatic Discharge curves at a rate of 1.9 mA/cm^2 , and B) a plot of the relative axial stress [%] vs displacement [mm] of the of the respective full cells. The rate applied was 6 mm per minute. C) Silicone molds used to fabricate electrodes with cavity sizes of different volumes. D) Fabrication steps to assemble the full cell. A detailed description is discussed in the experimental method.

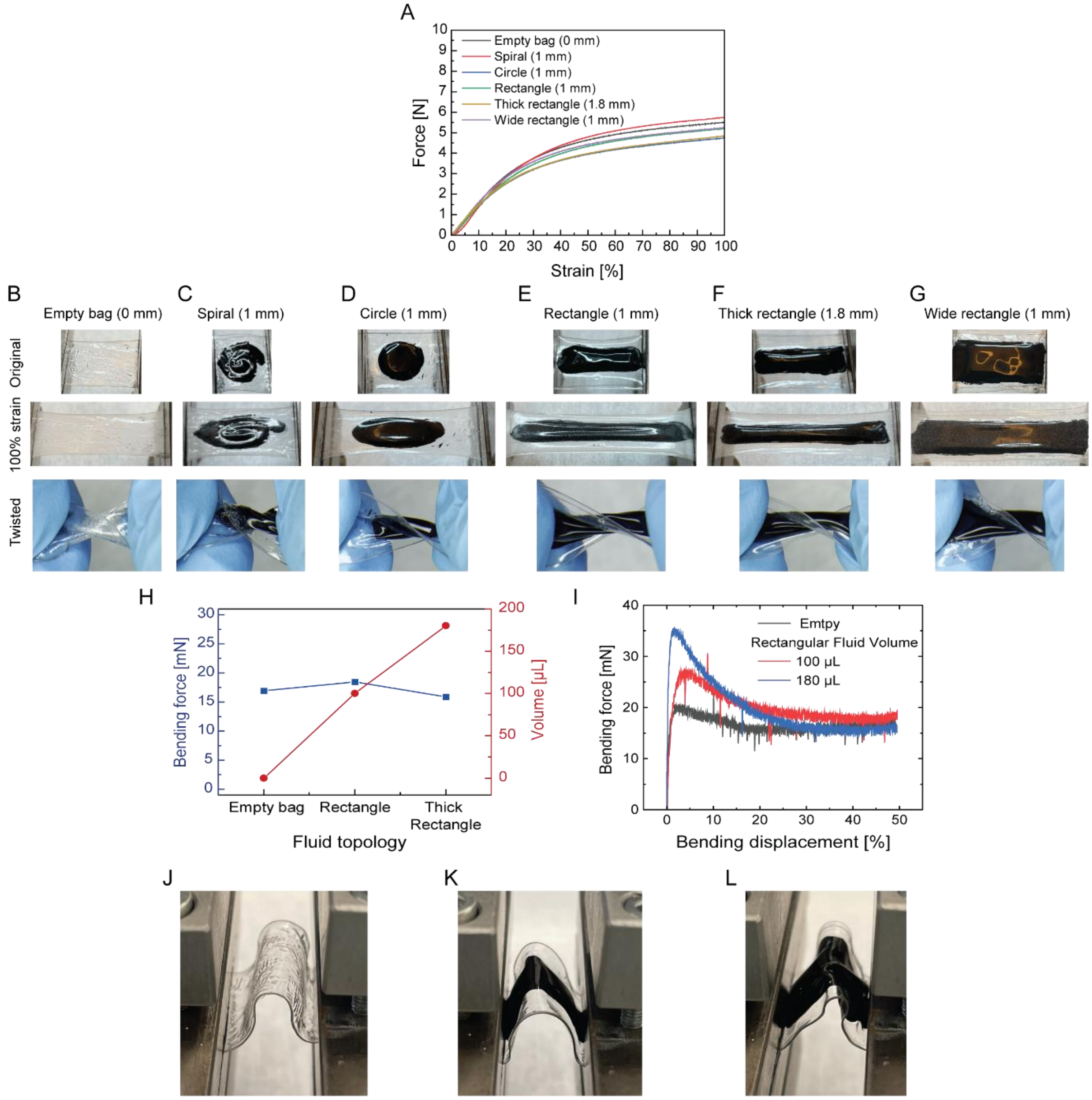

Figure S2. Fluid topology test. a) Force versus strain curve. Photograph images of the dummy cells under 100 % tensile strain and when twisted. The number in brackets indicates the cross-sectional thickness of the fluid. b) reference empty encapsulation bag without the fluid. The various fluid topologies sealed in the encapsulation bag. c) spiral, d) circle, e) rectangle, f) thick rectangle and g) wide rectangle. Fluid bending test. h) the relationship between bending force and fluids with different volume loading with a rectangular topology and i) corresponding measured bending force vs bending displacement. Photographs of the fluids in their bent state j) empty bag, k) rectangle (100 µL) and i) thick rectangle (180 µL).

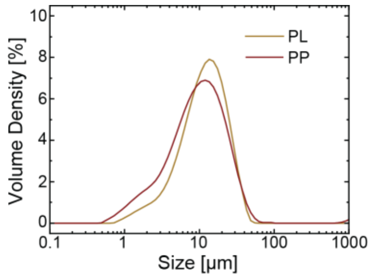

Figure S3. Particle size analysis of PL and PP performed in diluted deionized water instead of the acidic electrolyte (0.1 M $HClO_4$).

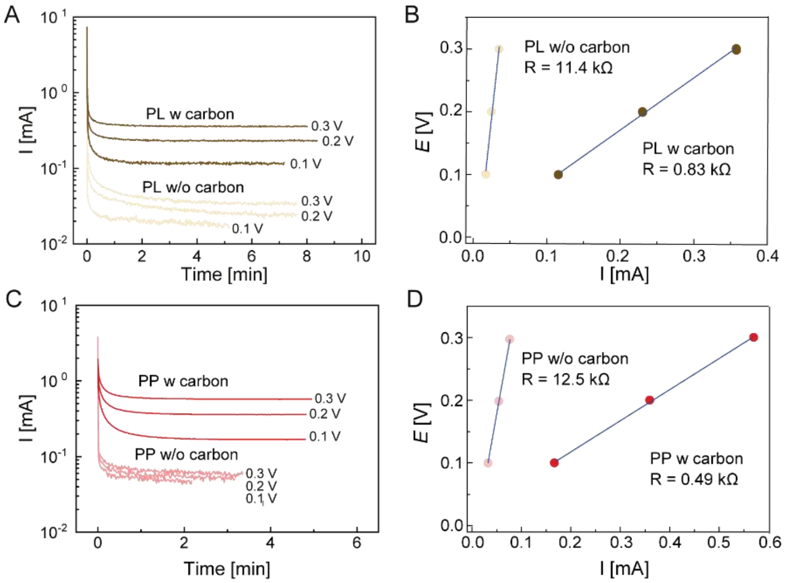

Figure S4. Electrical resistance of the fluids. The plots of current vs time and *V* vs *I* for PL (A and B) and PP (Cand D). To extract the resistance, the value of the current was taken at point where the current stabilizes. This is to remove contributions from ionic conductivity in the fluid. The blue line is the fitted slope to extract the resistance value.

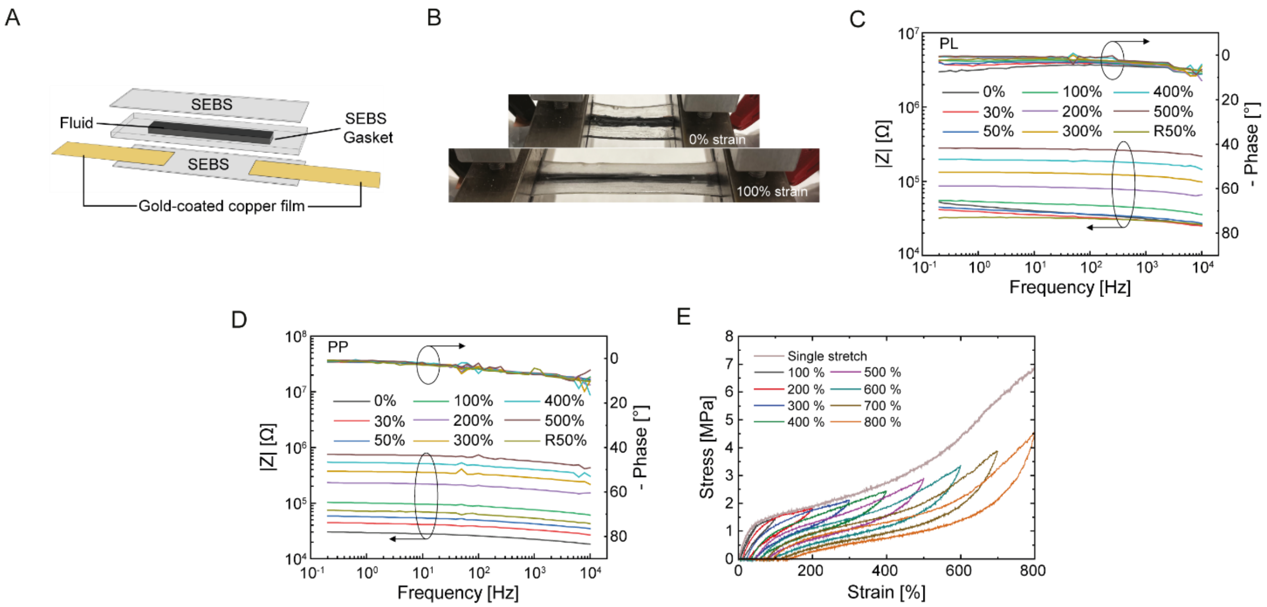

Figure S5. Electromechanical characterization of the redox-active electrofluids. A. Schematic of the experimental set-up showing the 2-point Au-coated Cu contacts with the SEBS encapsulation. B. Photograph of the device. Bode plots of taken from the electrochemical impedance spectroscopy measurements to extract the conductivity of the fluids. C. PL and D. PP. E. Force vs strain curves of a SEBS film measured at a single linear stretch and cyclic stretching at 100 % strain intervals until 800 % strain.

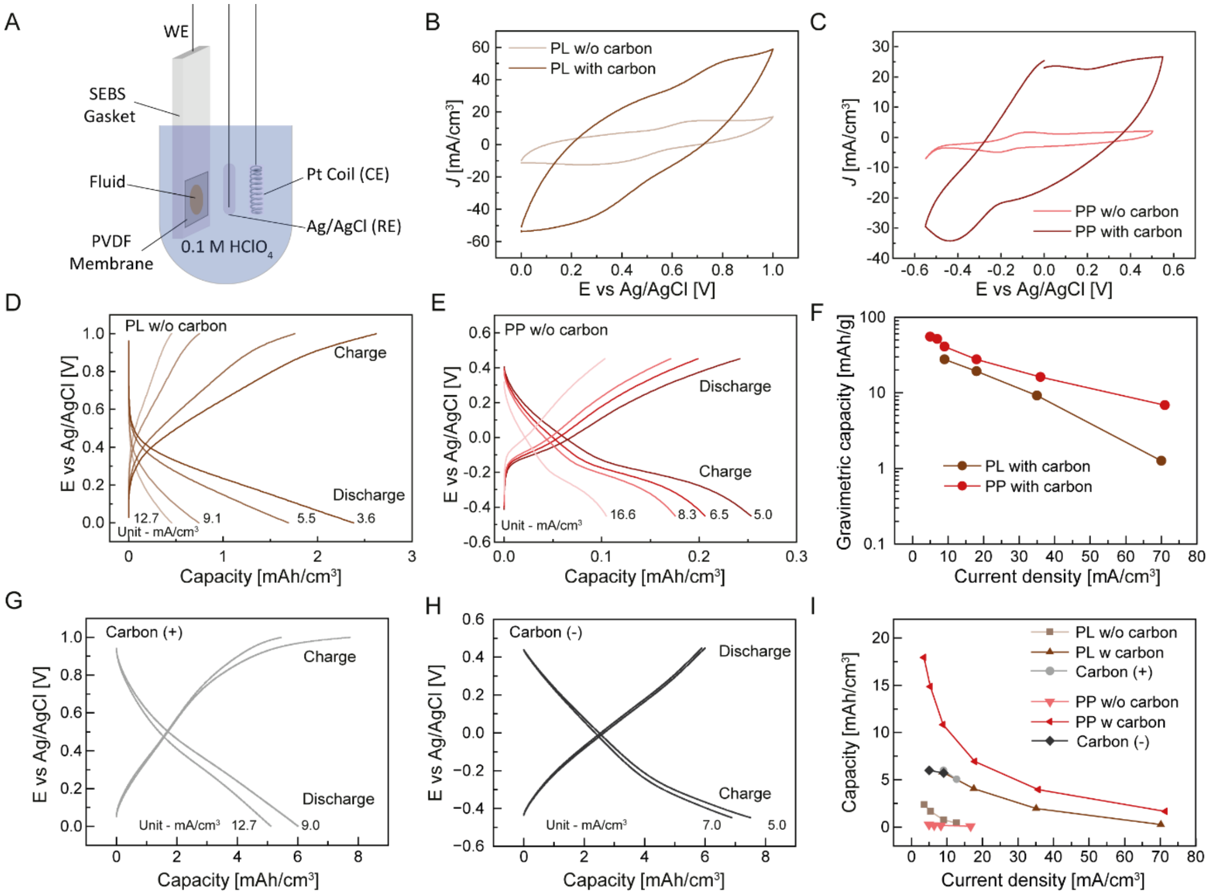

Figure S6. Electrochemical characteristics of fluids with and without carbon fillers. A Schematic of the half-cell. CV at 2 mV/s (B, C) and GCD (D ,E) of PL and PP respectively. F) Gravimetric capacity of PP and PL with carbon. GCD of the electrofluids containing the equivalent carbon fillers (activated carbon and carbon black) measured in (G) positive cathode (+) and (H) negative anode potentials. I) Comparison of the capacity contributions (vs current density) from the respective active particles in the fluids.

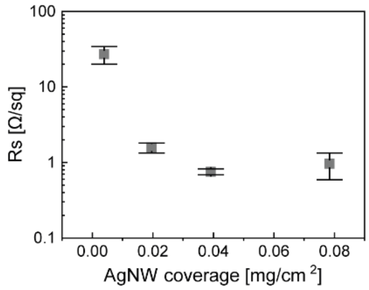

Figure S7. Optimisation of the AgNW coverage on the stretchable current collector. 0.03925 mg/cm^2 was selected since the Rs did not significantly increase with a larger coverage.

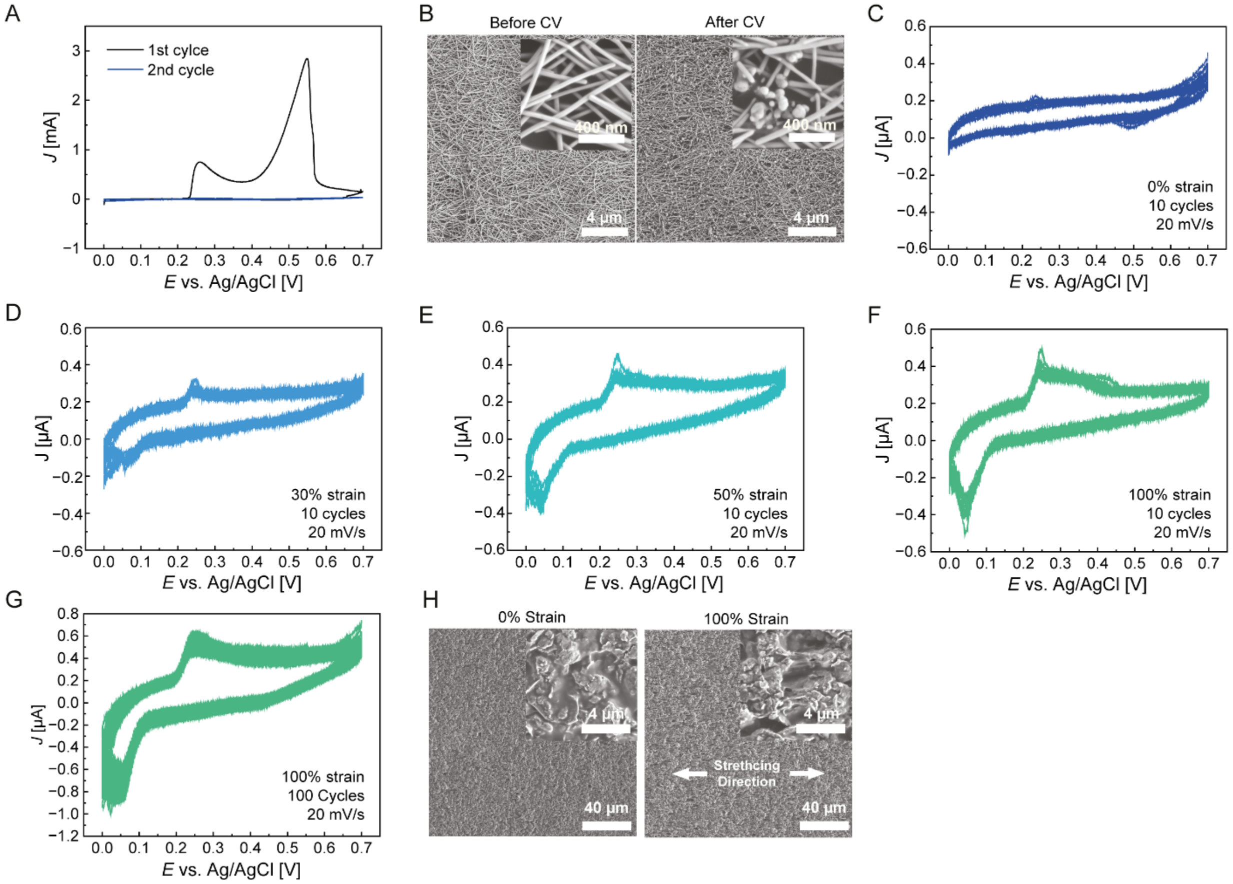

Figure S8. Electrochemical characterization of the current collector. A. Voltammograms of bare AgNW current collector in 0.1 M $HClO_4$ electrolyte. B. SEM image of the AgNW current collector before and after the CV scan. Voltammograms of the bilayer current collector (AgNW/NG:SEBS) at C. 0 %, D. 30 %, E. 50 %, and F. 100 % strain. G. 100 CV cycles at 100 % strain. H. SEM image of the bilayer current collector (AgNW/NG:SEBS) at 0 % (left) and 100 % strain (right).

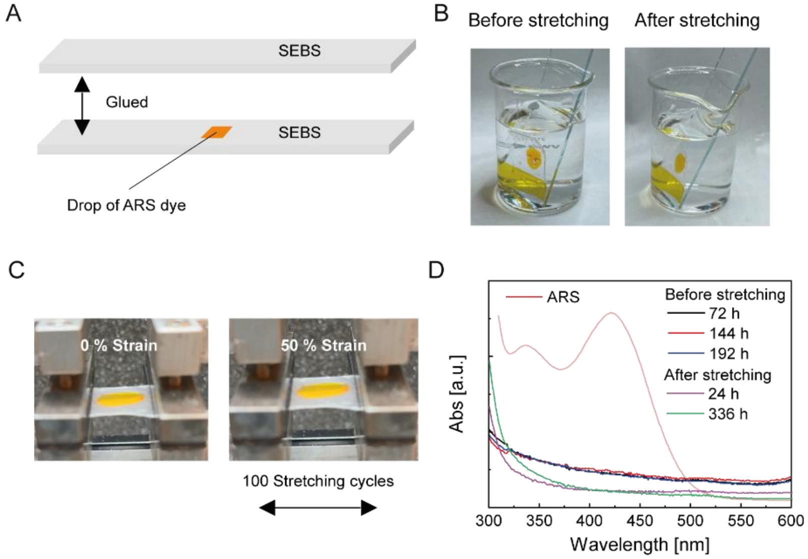

Figure S9. Stretchable encapsulation characterization. A. schematic of the SEBS bag. Photograph of the B. SEBS bag before and after stretching immersed in a beaker of the electrolyte, and C. SEBS bag being stretched. D. Absorption spectra of the electrolyte solution.

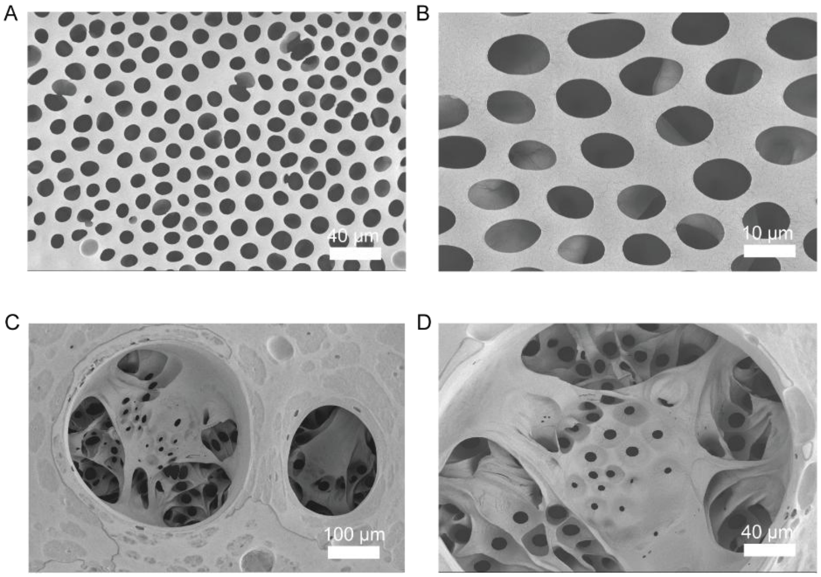

Figure S10. SEM analysis of the separator. A-B) top side of the membrane interfacing the air after the solvent evaporation process. C-D) Bottom side of the membrane interfacing the glass substrate.

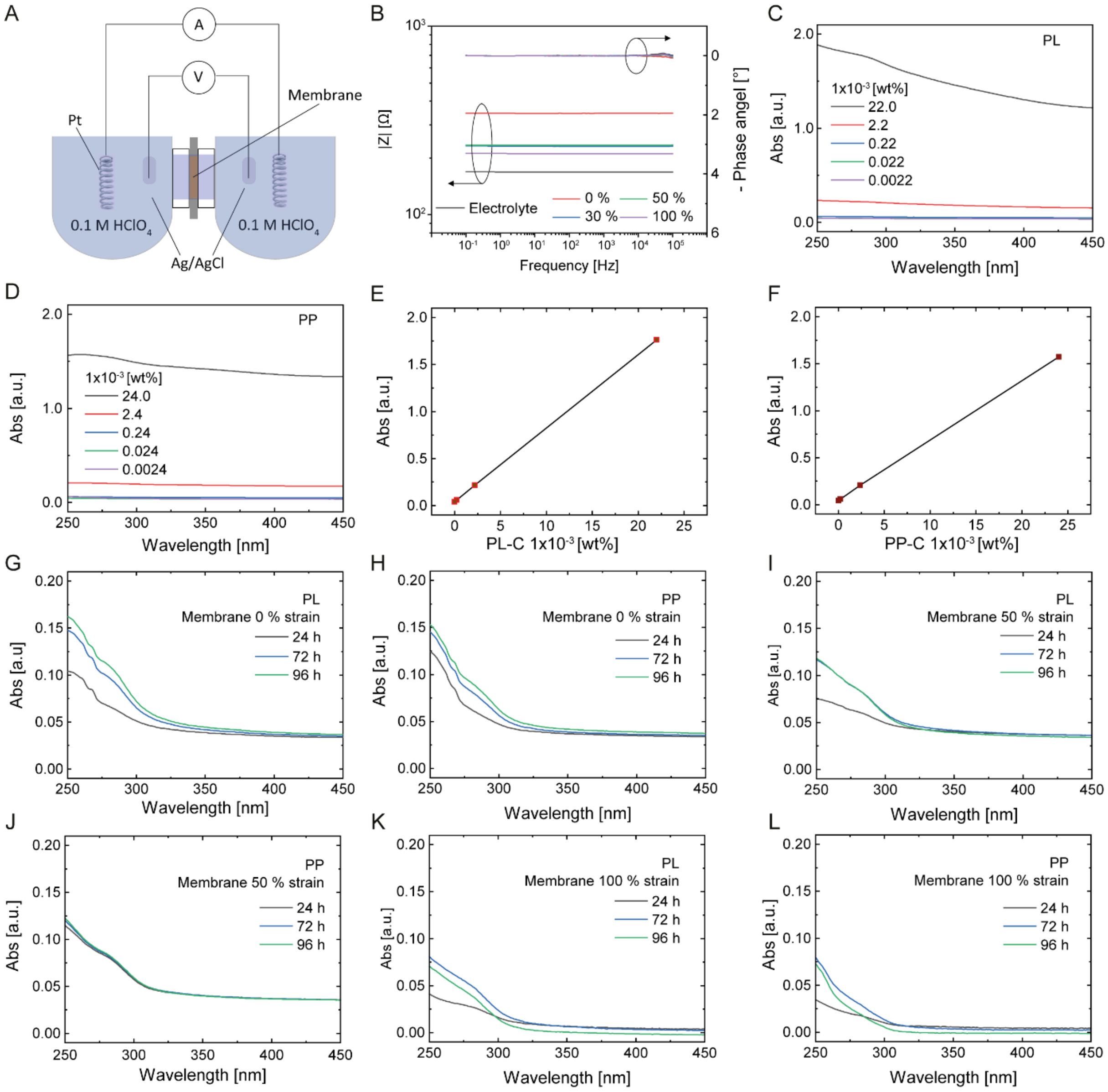

Figure S11. Membrane Characterization. A. Schematic of the H-cell used for membrane ionic conductivity experiments. Both unstretched and stretched membranes were sandwiched in between two rubber (SEBS) gaskets with an areal opening of 0.785 cm^2. Stretchable membrane separator characterization. B. Bode plots taken from the EIS measurements of the membrane at different strains. The Electrolyte used was 0.1 M $HClO_4$ (aq). Absorption spectra for the calibration curves for C, E) PL and D, F) PP, and the cross-over experiments at G, H) 0 %, I, J) 50 % and K ,L) 100 % strain.

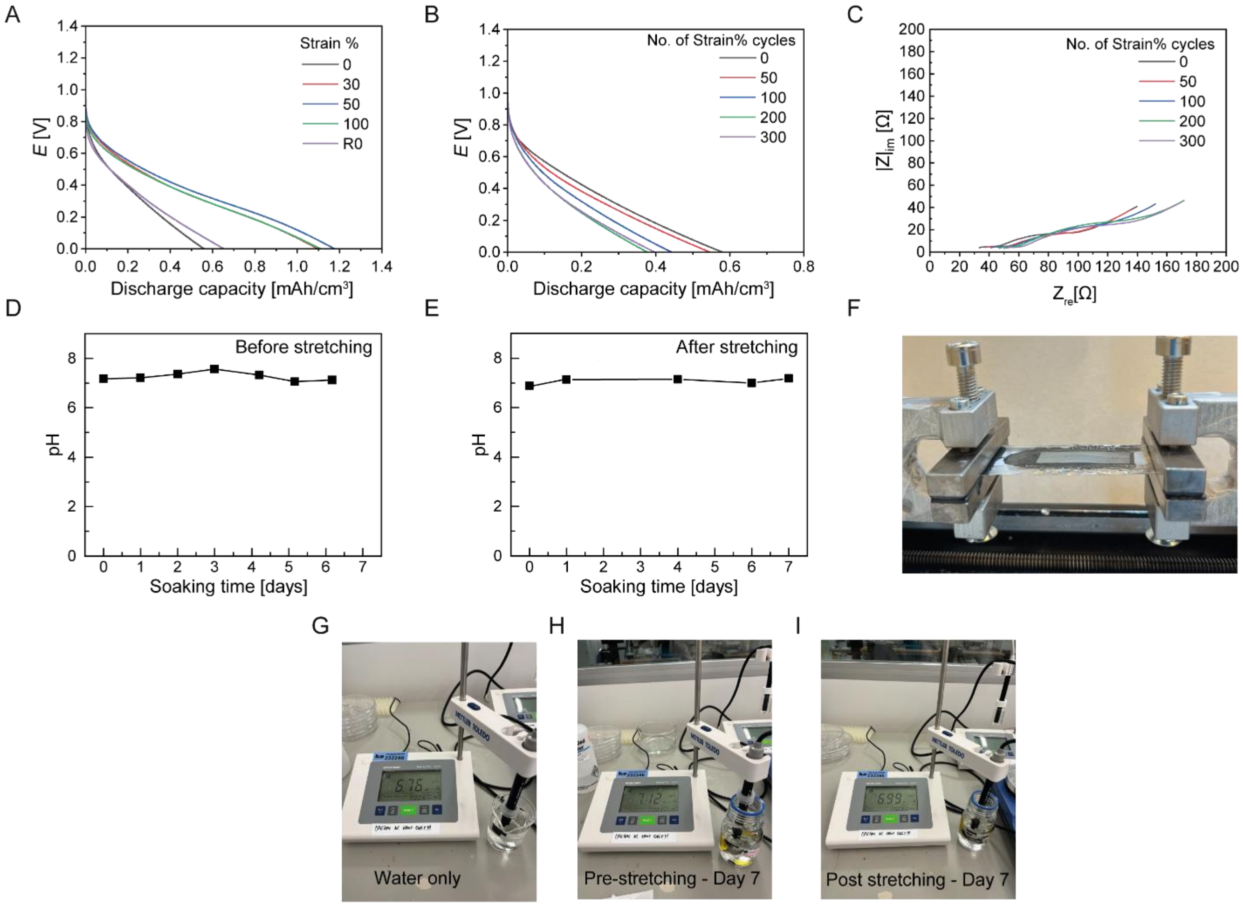

Figure S12. Electrochemical and mechanical characterization of the full cell. Galvanostatic discharge curves of the cell a rate of 1.9 mA/cm^2 measured at A. a specified strain (R0 = return), and B. after 50 cycles intervals at 30 % strain for a total of 300 strain cycles, and its corresponding C. EIS Nyquist plot. pH changes of the water containing the immersed cell D. before stretching over 6 days and E. after stretching at 30 % over 100 cycles over 7 days. Photographs of the F. cell stretched at 30 % strain, the pH meter measuring the G. water at day 0, and at day 7 H. before and I. after stretching the cell.

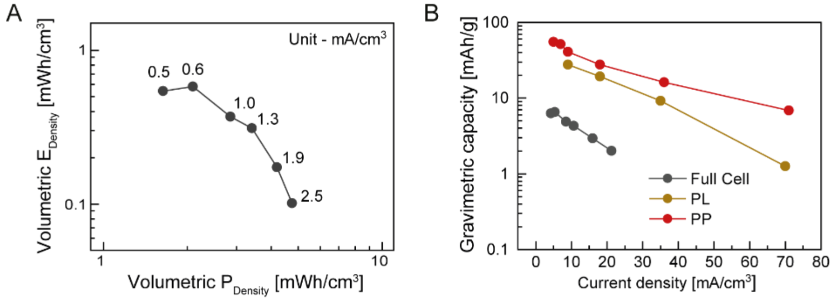

Figure S13. A. Ragone plot showing the specific volumetric capacities of the full cell and B. Gravimetric capacities of the full cell and the half-cell of PL and PP.

Legends for movies S1 to S3

Movie S1 - The redox-active electrofluid being extruded from syringe
Movie S2 - Full cell cut in half with fluid being squeezed out
Movie S3 - Battery powering LED under mechanical deformation
